# Omentin protects H9c2 cells against docetaxel cardiotoxicity

**DOI:** 10.1371/journal.pone.0212782

**Published:** 2019-02-22

**Authors:** Ricardo Lage, María Cebro-Márquez, Moisés Rodríguez-Mañero, José Ramón González-Juanatey, Isabel Moscoso

**Affiliations:** 1 Cardiology Group, Center for Research in Molecular Medicine and Chronic Diseases (CIMUS), Universidade de Santiago de Compostela and Health Research Institute, University Clinical Hospital of Santiago de Compostela—Santiago de Compostela, Spain; 2 Centro de Investigación Biomédica en Red de Enfermedades Cardiovasculares (CIBERCV), Madrid, Spain; 3 Department of Cardiology and Coronary Unit, University Clinical Hospital of Santiago de Compostela, Santiago de Compostela, Spain; National Institutes of Health, UNITED STATES

## Abstract

**Background:**

Association between obesity and cardiovascular diseases is well known, however increased susceptibility of obese patients to develop several cancer types is not so commonly known. Current data suggest that poorer overall survival in cancer patients might be associated to non-cancer-related causes such as higher risk of cardiotoxicity in obese patients treated with chemotherapeutic agents. Omentin, a novel adipokine decreased in obesity, is actually in the spotlight due to its favourable effects on inflammation, glucose homeostasis and cardiovascular diseases. Also, recent data showed that *in vitro* anthracycline-induced cardiomyocyte apoptosis is counteracted by omentin suggesting its cardioprotective role.

**Objective:**

Our aim was to evaluate omentin effects against docetaxel toxicity.

**Results:**

Our data indicate that omentin inhibits docetaxel-induced viability loss and that increased viability is associated to decreased caspase-3 expression and cell death. Although omentin reduces NOX4 expression, it failed to reduce docetaxel-induced reactive oxygen species production. Our results indicate that omentin decreases docetaxel-induced endoplasmic reticulum stress, suggesting that cardioprotective role might be associated to ERS inhibition.

**Conclusion:**

These data suggest that omentin treatment may contribute to decrease susceptibility to DTX-induced cardiotoxicity.

## Introduction

Obesity has become a worldwide epidemic, and its prevalence has been projected to grow by 40% in the next decade [1]. A follow-up analysis from the Framingham study established obesity as an independent risk factor for developing heart failure (HF), coronary artery disease (CAD), stroke, and overall cardiovascular disease (CVD) death, but is also associated with a higher prevalence of comorbidities such as diabetes, hypertension, and metabolic syndrome, which finally increase the risk for CVD [2].

Although the association between obesity and CVDs is widely known, increased cancer susceptibility of patients with obesity is not so commonly reported. Obesity has been strongly associated with cancer occurrence [3], shorter time to recurrence and with increased cancer-mortality [3, 4]. Current data indicate that poorer overall survival might be associated to non-cancer-related causes such as higher probability of cardiac adverse events in patients with obesity compared to lean subjects after treatment with chemotherapeutic agents [5].

Steady progress in anticancer agents development has led to a significant increase in patients survival from which has emerged the need to increase the knowledge of comorbidities and medical complications associated or caused by chemotherapy treatments. Adverse cardiovascular events are the major cause of morbidity and mortality in early-diagnosed breast cancer survivors. A recent meta-analysis indicates that overweight and obesity are risk factors for cardiotoxicity (CT) in breast cancer patients [6]. In addition, increased sensitivity to cardiac systolic impairment and cardiomyocyte mitochondrial dysfunctions have been demonstrated in murine models of obesity treated with anthracyclines [7, 8]. In opposition to the conventional perspective as a passive reservoir for energy storage, adipose tissue is actually recognized as an endocrine organ that expresses and secretes a variety of bioactive peptides, known as adipokines, with deleterious or beneficial effects on cardiovascular system. Recent data placed the altered endocrine function of adipose tissue in patients with obesity into the spotlight as a potential mechanism in the relationship between obesity and CT [9, 10]. Among the “good” adipokines, omentin (OMT) is actually attracting much attention due to its favourable effects on inflammation, glucose homeostasis and CVD. Low levels of OMT are linked to CAD, HF, acute myocardial infarction (AMI) and ischemic disease in patients with type 2 diabetes mellitus [11, 12]. In addition, regarding the mechanism of action at the molecular level of OMT, Kataoka *et al*. described that through AMPK and Akt signalling, OMT is able to protect cardiomyocytes from apoptosis ischemia/reperfusion injury [13]. It has been also reported that plasma OMT-1 levels are significantly decreased in patients with obesity [14]. Recent data showed that *in vitro* anthracycline-induced cardiomyocyte apoptosis is counteracted by OMT through the inhibition of oxidative stress suggesting that defective levels of OMT in obese subjects, in addition to obesity-related carcinogenesis, [15] might contribute to chemotherapy-induced CT [10]. Although more recently developed chemotherapy agents are emerging less cardiotoxic, it is being difficult to completely remove CT when using classic chemotherapy [16]. Docetaxel (DTX) is a second-generation taxane, effectively used against different types of cancers [17], that stabilizes the β-tubulin subunit of microtubules, preventing depolymerization of the mitotic spindle. Taxanes promote bradi- and tachyarrhythmias, myocardic ischemia and heart failure [18, 19]. At molecular level, taxanes are capable to activate several apoptosis pathways [17, 20]. Since OMT counteracts doxorubicin-induced apoptosis, the aim of our study is to evaluate the possible cardioprotective effects of OMT against DTX-induced apoptosis.

## Materials and methods

### Cell culture and reagents

Rat ventricular cardiomyoblast cells (H9c2) were used as an alternative to primary cardiomyocytes, these cells maintain morphological characteristics of immature embryonic cardiomyocytes with electrical and hormonal signal pathway elements of adult cardiac cells [21], in addition to energetic similarities to primary cardiomyocytes [22]. H9c2 were cultured in 0.1% gelatine coated plates with DMEM medium (Sigma-Aldrich, St. Louis, MO, USA) supplemented with 10% fetal bovine serum (FBS), antibiotics (100 UI/mL penicillin, 100 μg/mL streptomycin) and 2mM L-glutamine, in a 5% CO_2_ atmosphere at 37°C. H9c2 were seeded at least 24h before treatments unless otherwise indicated and the experimental procedures were conducted upon reaching 80% confluence.

Cells were treated with OMT (Sigma-Aldrich, St. Louis, MO, USA), N-Acetyl-L-cysteine (NAC) (Sigma-Aldrich) and DTX (Sigma-Aldrich, St. Louis, MO, USA) at indicated concentrations. Control groups were treated with respective culture medium and vehicle.

### Flow cytometry analysis of ROS

H9c2 cells were incubated during 24 hours with 25nM DTX and/or 300ng/ml OMT and/or 1h of pre-treatment with 4mM NAC in a 5% CO_2_ atmosphere at 37°C. H9c2 cell cultures were washed in PBS, trypsinized and resuspended in HBSS phenol-red-free medium (Sigma-Aldrich) at 5×10^5^ cells/ml. Samples were incubated at 37°C in the dark with 5μM dihydroethidium (DHE) for 30 min (Sigma-Aldrich). DHE is a specific biomarker of total superoxide anion (O_2_^−^). After incubation, cells were washed twice in cold PBS to eliminate excess staining solution. ROS (O_2_^−^) was measured by flow cytometry analysis of 5000-gated cells using FACScan and CellQuestPro software from Becton Dickinson (Fullerton, CA, USA) in channel FL2. The autofluorescence in each sample was subtracted.

### Cell viability

Cell viability was measured using the 3-[4,5-dimethylthiazol-2-yl]-2,5 diphenyltetrazolium bromide (MTT) assay (Sigma-Aldrich, St. Louis, MO, USA). Briefly, H9c2 cells were seeded at a density of 5000 cells/well in 96-well plates. Cells were treated with 25nM DTX and/or 300ng/ml OMT in a 5% CO_2_ atmosphere at 37°C. After, MTT (0.5 mg/ml) was added and incubated at 37°C during 4 hours. Formazan crystals were solubilized with dimethyl sulfoxide (DMSO) and isopropanol (1:1). The optical density (OD) was measured at a wavelength of 570nm and 690nm using an automated microplate reader.

### Flow cytometry of apoptosis

Apoptosis was measured by using FITC Annexin-V-FLUOS staining Kit (Roche Diagnostics) according to the manufacturer´s protocol. H9c2 cells were incubated during 24 hours with 25nM DTX and/or 300ng/ml OMT in a 5% CO_2_ atmosphere at 37°C. Then, cells were collected by trypsinization and centrifuged at 1200 rpm for 5 minutes. Following suspension in binding buffer, cells were labelled with Annexin-V-FITC and Propidium Iodide (PI) according to the manufacturer's instructions. FITC and PI were measured by flow cytometry analysis of 5000-gated cells using FACScan and CellQuestPro software from Becton Dickinson (Fullerton, CA, USA). Cells populations were defined as necrotic (PI+), apoptotic (AV+/PI+) or early apoptotic (AV+) cells.

### Real-time quantitative PCR

Total RNA was isolated from cell culture using *TRI Reagent* (Sigma-Aldrich, St. Louis, MO, USA), according to the manufacturer’s recommendations. First-strand cDNA was synthesized from 1μg total RNA using *RevertAid First Strand cDNA Synthesis Kit* (Thermo Scientific, MA, USA), according to the manufacturer’s recommendations. The resulting cDNA was subjected to Real-time PCR using *FastStart Universal SYBR Green Master (Rox)* (Roche Molecular Biochemicals, Mannheim, Germany) was performed using specific primers (S1 Table). All reactions were carried out in an *Eppendorf Realplex termoclycler*. Sample values were standardized versus an indicated housekeeping gene.

### Western blot

H9c2 cells were homogenized in ice-cold lysis buffer containing 50 mmol/l Tris-HCl, pH 7.5, 1 mmol/l EGTA, 1 mmol/l EDTA, 1% Triton X-100, 1 mmol/l sodium orthovanadate, 50 mmol/l sodium fluoride, 5 mmol/l sodium pyrophosphate, 0.27 mol/l sucrose, 0.1% 2-mercaptoethanol, and 1x *Complete Protease Inhibitor Cocktail* (Roche Diagnostics, Mannheim, Germany) for 1 hour. Homogenates were centrifuged at 21.100xg for 15 min at 4°C; supernatants were removed, and frozen at -20°C. Cells lysates (15μg) were subjected to 13% acrylamide SDS-PAGE gels, transferred on a PVDF membrane as previously described. Membranes were blocked for 1 hour in TBS-Tween (TBST: 50mmol/l Tris-HCl, pH 7.5, 0.15mol/l NaCl and 0.1% Tween) containing 3% BSA and probed with primary antibodies (S2 Table). Detection of proteins was performed using appropriate secondary antibody (S2 Table) and an enhanced chemiluminescence reagent *Pierce-ECL western blotting substrate* (Thermo Scientific, MA, USA).

Representative blots correspond to unedited images of full radiographic film detection. Each band is accompanied by its respective load control.

Densitometry analysis was performed using *ImageJ-1*.*33* software (*NIH*, Bethesda, MD, USA). First, each blot is relativized to a background value obtained from an identical adjacent area to consider any background variation in the radiographic signal. Each blot is then individually relativized versus its control to avoid any load variation between samples. Mean of 3 samples was used as experimental group value for each single experiment (n = 3).

### Statistical analysis

Data are represented as mean ± SEM (standard error of the media). Statistical significance was determined by Mann-Whitney, ANOVA followed by Tukey´s or Dunn´s post hoc test or two-way Student’s t-test determined by using *GraphPad Prism 6 Software*. P<0.05 was considered significant.

## Results

### Effects of DTX and OMT treatment in H9c2 cell viability and apoptosis

**Fig 1:**
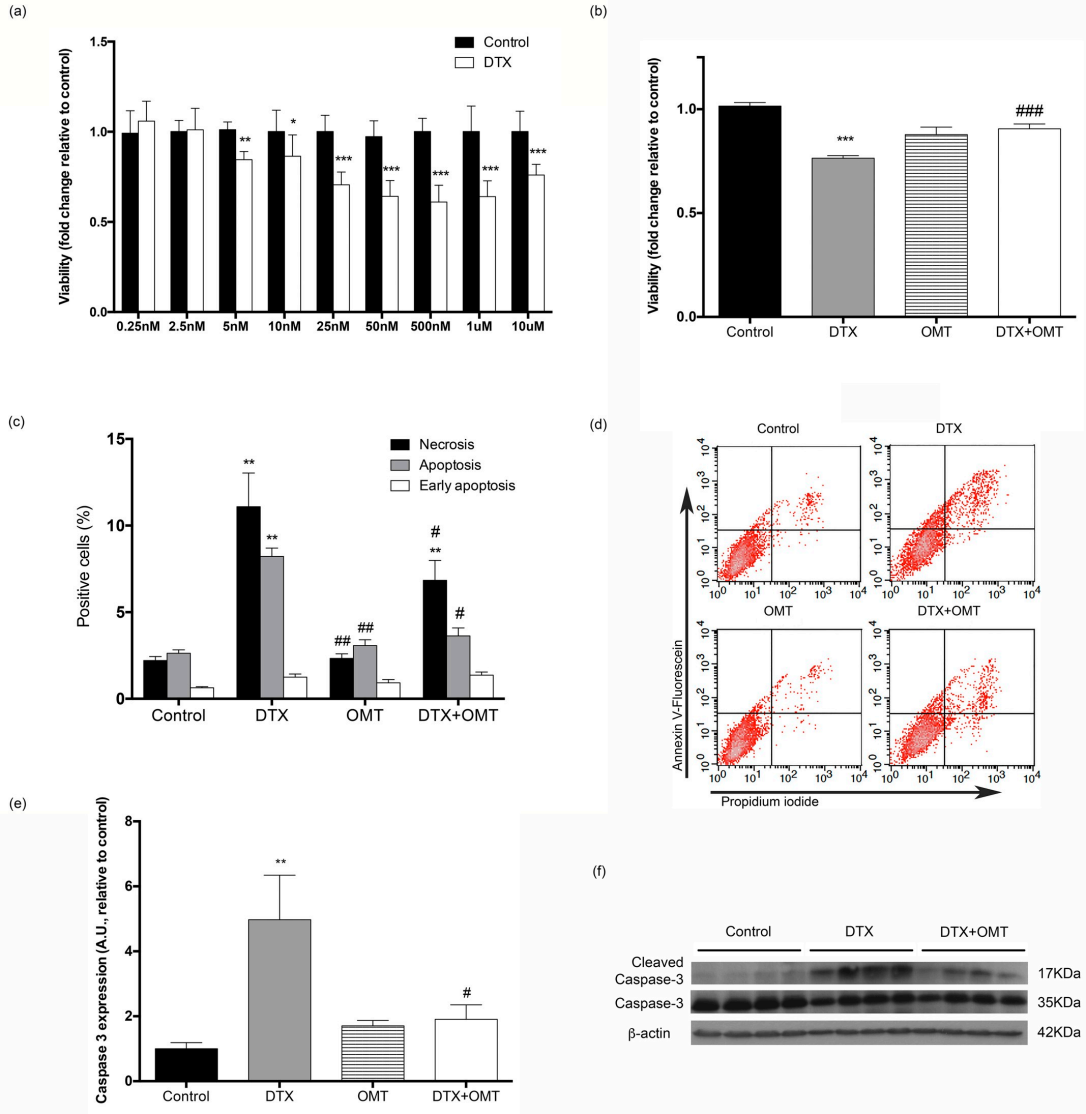
Effect of OMT in viability, apoptosis and Caspase 3 expression in H9c2 cells exposed to DTX. (A) Histograms showing cell viability measured by MTT assay in H9c2 cell treated with DTX after 24 h. (B) MTT cell viability assay in H9c2 cells treated with DTX and/or OMT after 24 hours. (C) Annexin V flow cytometry assay on H9c2 cell treated with DTX and/or OMT. (D) Representative density-plots images of Annexin V-FITC/propidium iodide (PI) double staining flow cytometry. (E) Caspase-3 levels in H9c2 cells treated with DTX and/or OMT after 24 hours. (F) Representative western blot autoradiographic images. Data are expressed as mean ± SEM of three or four independent experiments. Statistical significance *, ** and ***p < 0.05, 0.01, and 0.001 vs. control; #, ##, ### p< 0.05, 0.01 and 0.001 DTX-OMT vs. DTX.

Viability of H9c2 cardiomyoblast cells was evaluated after 24h exposure to different concentrations of DTX by using MTT assays. As shown in Fig 1A, docetaxel decreased H9c2 cell viability in a dose-dependent manner. Based on the extremely significant decrease of the viability (Control; 1.00 ± 0.03, n = 8 vs. DXT; 0.71 ± 0.25, n = 8, Fig 1A), 25nM of DTX was selected as cardiotoxic concentration. H9c2 cells were co-treated with 300 ng/ml during 24h based on previously described antiapoptotic role of omentin [23]. OMT counteracted DTX-induced decrease in H9c2 viability (DTX; 0.76 ± 0.13, n = 3 vs. DTX+OMT (300 ng/ml); 0.97 ± 0.11, n = 3, Fig 1B).

Flow cytometry apoptosis assay showed that the treatment of H9c2 cells with DTX increases necrosis and apoptosis compared to control group (Fig 1C and 1D). After 24 hours of OMT co-treatment both necrosis (Control; 2.21 ± 0.23, n = 7 vs. DXT; 11.09 ± 1.95, n = 7 vs. DTX+OMT; 6.84 ± 1.15, n = 7) and apoptosis (Control; 2.62 ± 0.28, n = 7 vs. DXT; 8.22 ± 0.48, n = 7 vs. DTX+OMT; 3.63 ± 0.46, n = 7) have been significantly reduced. Early apoptosis was not affected after any of the treatments (Fig 1C and 1D).

We next examined the effects of OMT co-treatment on DTX-induced caspase-3 activation (ratio 17KDa/35KDa) as a major effector of cell death. H9c2 cardiac cells were co-treated with OMT (300 ng/ml, 24h) and DTX. OMT counteracted caspase-3 activation induced by DTX (DTX; 4.97 ± 1.37, n = 3 vs. DTX+OMT; 1.91 ± 0.44, n = 3, Fig 1E and 1F).

### Effects of OMT on DTX-induced ROS production, NADPH oxidases, CAT, GPx and SOD1 expression in H9c2 cells

**Fig 2:**
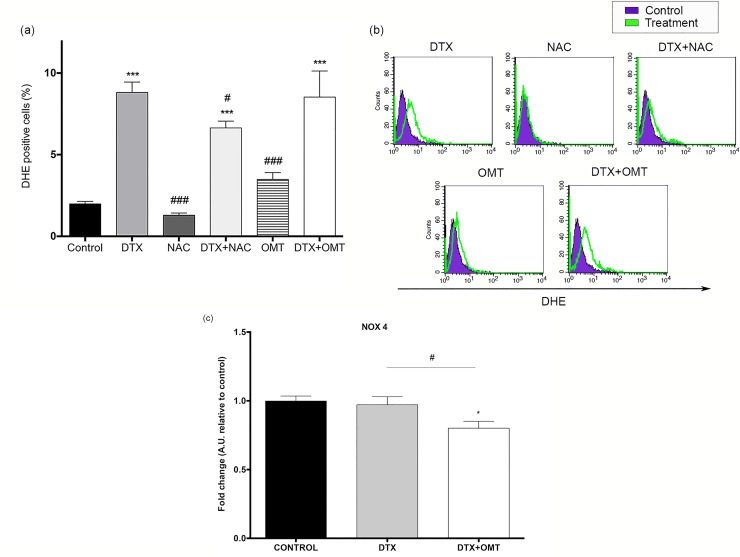
Effect of OMT in ROS production and NADPH oxidase (NOX4) expression in H9c2 treated with DTX. (A) ROS levels determined by flow cytometry using DHE staining in H9c2 cells treated with DTX and/or OMT after 24 hours and/or pre-treated with NAC for 1h. (B) Representative histogram images of DHE staining. (C) Histograms showing NOX4 mRNA expression measured by real-time polymerase chain reaction in H9c2 cell treated with DTX after 24 h. Data represent the means ± SEM from at least three independent experiments. Statistical significance *, ** and ***p < 0.05, 0.01, and 0.001 vs. control; #, ##, ### p< 0.05, 0.01 and 0.001 DTX-OMT vs. DTX.

Intracellular ROS levels were assessed by DHE in H9c2 cell treated with DTX and OMT. While NAC supplementation significantly reduced ROS levels (NAC; 1.29 ± 0.14, n = 3 vs. DTX; DTX+OMT; 6.64 ± 0.42 vs control and DTX, n = 3, Fig 2A and 2B); OMT co-treatment does not affect DTX-induced ROS levels (DTX; 8.81 ± 0.64 relative to control, n = 3 vs. DTX+OMT; 8.53 ± 1.60, n = 3, Fig 2A and 2B).

Previous data showed that DTX increase endothelial ROS formation through NOX4 [24]. However our results failed to show increased expression of studied NOX isoforms (NOX1, NOX2 and NOX4) in DTX-H9c2 treated cells. Curiously, our results showed a marked decrease in NOX4 expression in co-treated cells (DTX; 0.97 ± 0.06 vs. DTX+OMT; 0.80 ± 0.05, Fig 2C) but any of the other analysed isoforms showed a regulation of gene expression after co-treatment (data not shown). Our results suggest that increased ROS levels are not directly associated with NOX isoforms expression.

**Fig 3:**
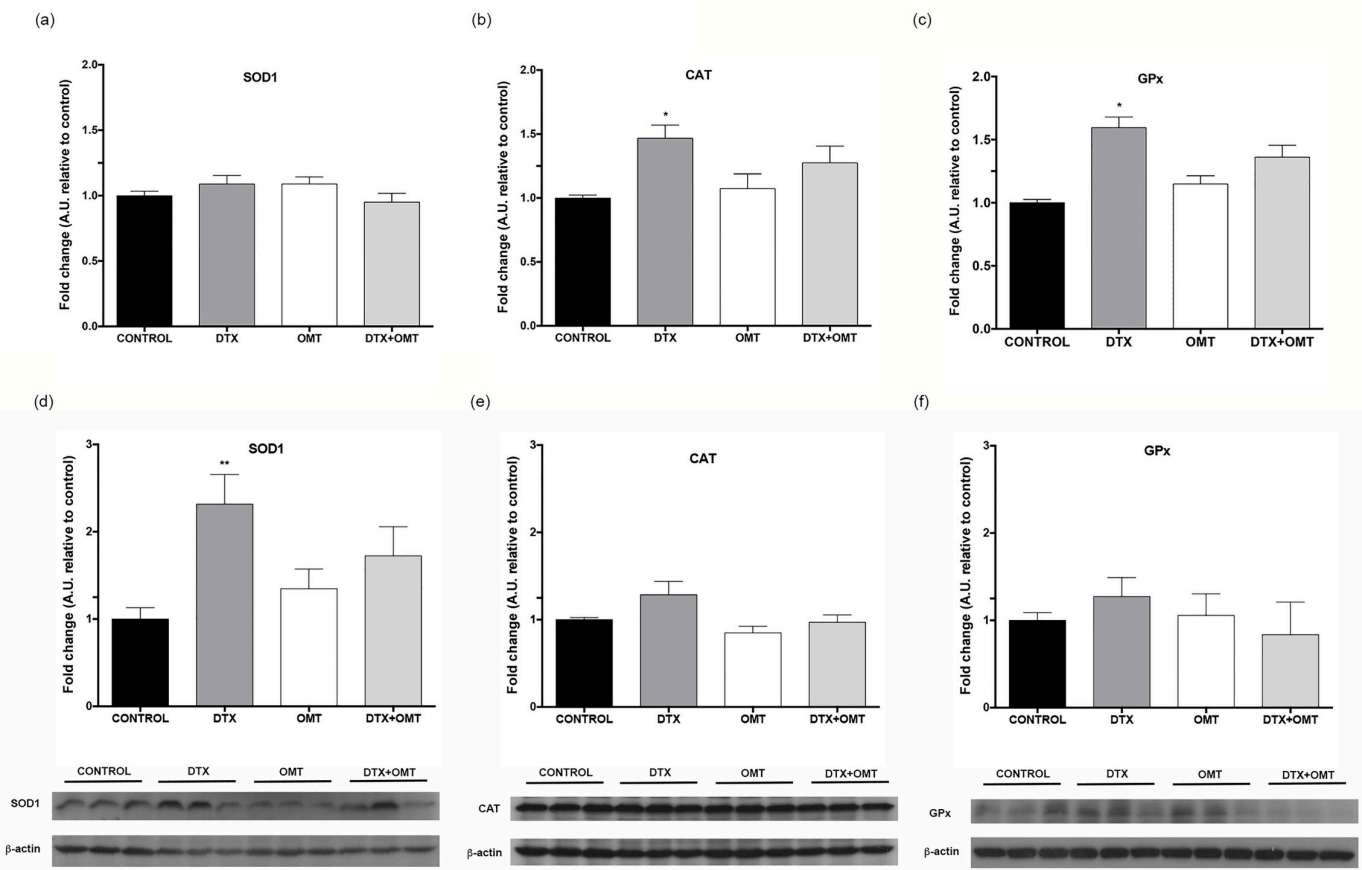
Effect of OMT in CAT, GPx and SOD1 antioxidant enzymes gene and protein expression in H9c2 treated with DTX. mRNA levels of (A) CAT, (B) GPx and (C) SOD1 determined by real-time RT-PCR and protein levels of (D) CAT, (E) GPx and (F) SOD1 determined by Western blot in H9c2 cells treated with DTX and/or OMT after 24 hours. Data represent the means ± SEM from at least three independent experiments. Statistical significance *, ** and ***p < 0.05, 0.01, and 0.001 vs. control; #, ##, ### p< 0.05, 0.01 and 0.001 DTX-OMT vs. DTX.

Because DTX-induced toxicity is known to disturb the intracellular redox balance and to cause oxidative stress in endothelial cells [24], antioxidant enzyme expressions including superoxide dismutase 1 (SOD1), catalase (CAT) and glutathione peroxidase (GPx) were determined. As shown in Fig 3, statistical differences were found after DTX treatment in CAT and GPX gene expression, but failed to show any difference in SOD1 expression. (Fig 3A–3C). We have also analyzed SOD1, CAT and GPX protein expression; surprisingly we only found statistical differences in SOD1 expression after DTX treatment (Fig 3D–3F). Catalase, SOD1 and glutathione levels were not affected by OMT supplementation in H9c2 cells (Fig 3A–3F).

### Effects of OMT on DTX-induced endoplasmic reticulum stress (ERS)

**Fig 4:**
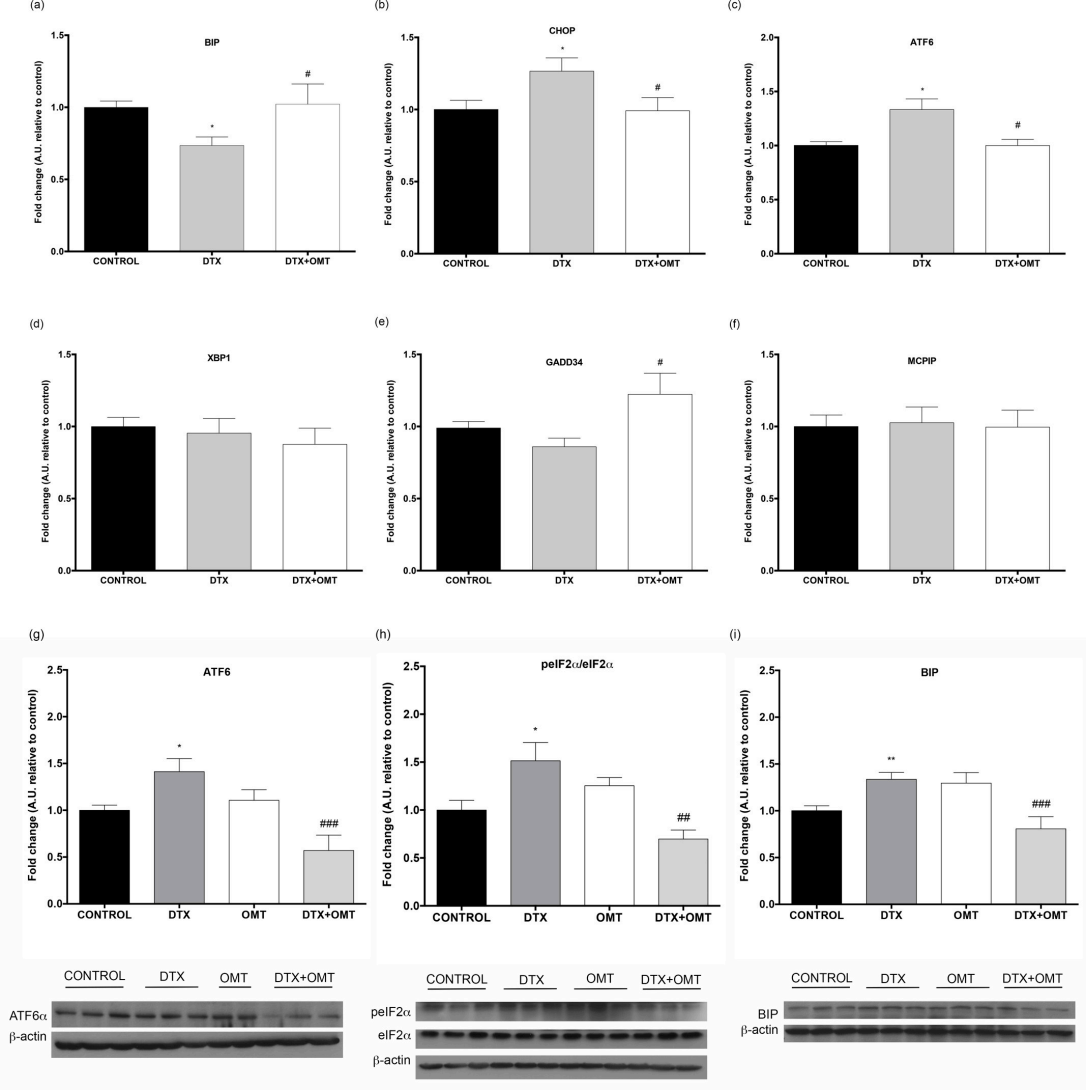
Effect of OMT in ERS gene and protein expression in H9c2 treated with DTX. mRNA levels of (A) BIP, (B) CHOP, (C) ATF6, (D) XBP1, (E) GADD34 and (F) MCPIP determined by real-time RT-PCR. Protein levels of (G) ATF6α, (H) peIF2α/eIF2α ratio and (I) BIP determined by western blot. All experiments were performed in H9c2 cells treated with DTX and/or OMT after 24 hours. Data represent the means ± SEM from at least three independent experiments. Statistical significance *, ** and ***p < 0.05, 0.01, and 0.001 vs. control; #, ##, ### p< 0.05, 0.01 and 0.001 DTX-OMT vs. DTX.

Due to the fact that ERS is one of the possible molecular mechanisms of cardiac toxicity [25, 26], we studied whether DTX induces ER stress in our *in vitro* model. Our results showed significantly decreased expression of BIP in addition to increased expression of ATF6 and CHOP indicating that DTX triggers ERS. We next examined if decreased cell death observed in H9c2 cells co-treated with DTX and OMT might be mediated by a reduction in ERS. Our data showed that co-treatment with OMT counteract DTX-induced regulation of ERS genes. OMT co-treatment, in addition to decrease CHOP and ATF6 and increase BIP expression, is capable to induce overexpression of GADD34 (n = 3, Fig 4A–4F). We have also analysed protein, according to gene expression analysis, we found that DTX-induced overexpression of ATF6α, peIF2α/eIF2α and BIP is counteracted by OMT (n = 3, Fig 4G–4I).

Our data suggest that ERS relieve is not associated with quenching ROS excess by omentin since co-treatment, in our conditions, counteracts ERS but does not reduce ROS levels. However, we wanted to assess this point by using pharmacological ROS scavenger NAC. Our data show that reduction of oxidative stress associated with NAC treatment reduces gene expression of CHOP and protein of ATF6 induced by DTX, but not the other previously described ERS proteins (n = 3, S1A–S1I Fig).

## Discussion

Worldwide obesity prevalence is estimated to reach 40% during the next decade. In addition to contributing to cardiovascular disease, obesity has been strongly associated with increased cancer incidence, particularly in postmenopausal women [3]. Although molecular mechanisms linking obesity and cancer risk/prognosis are not fully elucidated, obesity creates a host environment characterized by low-grade systemic inflammation, disturbed sex steroid signalling and increased insulin/insulin-like growth factors that may promote malignant cell growth and progression. Obesity is also associated with shorter time to disease recurrence, greater mortality [3, 4] and is an important prognostic factor of cardiotoxic events independent of treatment dose [27]. Recent data suggest that increased mortality in cancer patients might be associated to non-cancer-related causes of death such as higher probability of cardiac adverse events in patients with obesity treated with cardiotoxic agents [5].

Association between obesity and CVDs is well known for a long time, however increased susceptibility of patients with obesity to suffer chemotherapy-induced adverse cardiac events was not well documented until recently. An increased risk of CT as well as higher sensitivity to systolic alterations and mitochondrial dysfunction associated to obesity has been demonstrated in the last years, both in murine models [7, 8] and patients [6], respectively.

Although CT is not easily quantifiable, since most of available data comes from retrospective studies and only in a few cases a prospective evaluation of the cardiac function has been performed, CT is identified as one of the most common complications of current cancer therapies [28]. CT was commonly associated with the use of anthracyclines; however, lately breast cancer cytostatic drugs have also been associated to cardiovascular toxicity [28]. Recent studies suggest possible mechanisms by which CT trigger HF depending on the antineoplastic drug, focusing the origin of CT in oxidative stress-induced cardiomyocyte apoptosis [29]. Cumulative data suggest that mitochondrial cell damage is due to the formation of free radicals and increased oxidative stress, decreased ATP levels, decreased expression of SERCA and ERS induction, structural and functional damage that finally triggers cardiomyocyte death.

Several potential mechanisms such as overdose due to the adjustment to the real weight of the patient [30] or genetic background [31], which also might explain significant co-occurrence of CVD with multiple types of cancers independently of behavioural risk factors [32] have been suggested in the relationship between obesity and CT. Among them, changes in the endocrine function of adipose tissue and circulating adipokine levels associated with obesity seem to play a key role increasing the susceptibility to develop CT [9, 10].

Recent data showed that *in vitro* anthracycline-induced cardiomyocyte apoptosis is counteracted through the inhibition of oxidative stress by OMT [10], an adipokine released by visceral depots and down-regulated in patients with obesity [14]. Several studies show a tight relationship between circulating OMT levels and cardiovascular health. Low levels of OMT are linked with CAD, HF, AMI and ischemic disease in patients with type 2 diabetes mellitus [11, 12]. Previous reports have demonstrated a protective effect of OMT as therapeutic agent for CVD [33]. As it was previously reported, OMT reduces apoptosis in H9c2 treated with doxorubicin [10], it also reduces apoptosis in rat cerebral ischemic conditions [34] and in mice subjected to myocardial ischemia followed by reperfusion [13]. In HUVEC cells, OMT increased differentiation into vascular-like structures and decrease apoptotic activity under conditions of serum starvation [35].

In this study, we demonstrated that OMT significantly reduces DTX-induced both necrosis and apoptosis in H9c2 cardiomyoblasts, as it was shown with doxorubicin [10]. Our data demonstrated a noticeable increase in oxidative stress and ERS in H9c2 cardiomyoblasts treated with DTX, accompanied by a pronounced decrease in cell viability, as it was previously described [24–26]. DTX induced caspase-3-dependent apoptosis was previously described in human prostate cancer cell line [36], human oral squamous cell carcinoma cell lines [37], breast and ovary carcinoma cells [38], but to the best of our knowledge, this is the first time that direct action of DTX on H9c2 cells is associated with increased caspase-3 activity and increased cell death.

Oxidative stress plays a central role in cardiomyocyte death in several adverse cardiovascular events [39]. NADPH oxidases (NOX) are the major source of O^2–^ production, NADPH oxidase 2 (NOX2) and 4 (NOX4) are both expressed in cardiac muscle. It has been previously described that upregulation of NOX4 by hypertrophic stimuli increases apoptosis and mitochondrial dysfunction in cardiac myocytes. NOX4 has also been demonstrated to be an important source of oxidative stress in the failing heart [40]. Previous data demonstrated that DTX treatment increased NOX activity in HUVEC cells resulting in ROS formation [24] whereas OMT inhibits NOX activity in vascular smooth muscle cells after tumour necrosis factor alpha and platelet-derived growth factor treatment [23, 41]. We therefore studied whether DTX might also caused an increase in oxidative stress that justify increased cardiomyocytes death and whether OMT could also counteract the increase of stress as in vascular smooth muscle cells. Our data demonstrated a significant increase in ROS formation in H9c2 cells treated with DTX but failed to show any improvement after treatment with OMT despite the significant decrease in NOX4 expression; NOX1 and NOX2 expressions were not affected in our experimental model (data not shown). Although OMT may influence activity of alternative endogenous anti-oxidative enzymes, including super oxide dismutase, catalase and glutathione peroxidase [10], our data indicate that the decrease in cell death is not associated to a lower oxidative stress since OMT does not counteract the increase in ROS levels. Oxidative stress, caused by the overproduction of ROS and the decrease in the antioxidant level, is prevented or suppressed by major antioxidant enzymes, including SOD, CAT and GPx. In our study, DTC treatment increases CAT, GPX and SOD1 gene expression, probably as a compensatory response against higher ROS levels or previously described reduced enzymatic activity associated to DTX treatment [42–44]. However, only in the case of SOD1 protein levels that increase is significant. Coherently with the lack of omentin effect on oxidative stress, OMT did not play any role on the expression of antioxidant enzymes in our model. Our data suggest that OMT exerts its protective role through another pathway of damage.

Currently, ERS-mediated cardiomyocyte apoptosis has attracted broad attention, because it has been found to be responsible for the pathophysiology of many CVDs [45]. The ER is an organelle responsible of the folding of secretory and membrane proteins. Different stimuli cause the accumulation of unfolded and misfolded proteins, dissociating BIP from the three ER transmembrane protein sensors PERKS, ATF6 and IRE1, triggering the unfolded protein response (UPR) [46]. It has been recently published that after a strong or prolonged stimulus, CHOP, caspase-12, and/or JNK-dependent apoptotic signalling pathways are activated. Thus, CHOP apoptotic pathway activates caspase-3 related with Bcl-2 family proteins [45]. Previous data demonstrated that DTX-induced apoptosis is mediated by induction of ERS [25]. Consequently we analysed the expression of genes involved in ERS such as BIP, CHOP, ATF6, XBP1, GADD34 and MCPIP in H9c2 DTX-treated cells and the effect of OMT co-treatment in ERS gene expression. Although, conventionally ER stress response triggers upregulation of BIP [46], our results showed a significant downregulated BIP expression counteracted by OMT. However, according with conventional ERS response BIP protein levels increase after DTX treatment and decrease after OMT/DTX cotreatment. CHOP is a molecule involved in ER stress-induced apoptosis, under non-stressed conditions CHOP expression is low, but increases in response to ER stress through IRE1-, PERK- and ATF6-dependent transcriptional induction [47]. In addition, it has been proposed that ERS signalling, especially mediated by ATF6, induces XBP1 [48] and plays a decisive role in the induction of apoptosis in muscle tissues through activation of caspase-12 and subsequently activated caspase-3 [49]. Our results showed a significant DTX-induced ATF6 upregulation, according to previous data in human melanoma cells [25], accordingly to these results protein analysis also shows an increase in ATF6α expression after DTX treatment and a decrease after OMT/DTX treatment, and a significant increase in CHOP expression both counteracted after OMT treatment. Increased ATF6 expression is accompanied by a slight, not significant increase, in XBP1 levels that is also inhibited by OMT. Coherently with the role of the DTX as trigger of ERS and according with previous data demonstrating that GADD34 is regulated by DTX in HL60 cells after 24 hours [50], our data showed a marked decrease in GADD34 expression that is recovered when H9c2 are co-treated with OMT. As it was previously shown in prostate cancer cells, DTX treatment in H9c2 cells also increases peIF2α/eIf2α protein expression, that is reduced after OMT co-treatment [25, 26, 51]. In addition, our data showed also a non-significant increase in the expression of the monocyte chemotactic protein-induced protein (MCPIP) inhibited by OMT. Based on previous data, MCPIP up-regulation is probably associated with a previously described increase in the expression of MCP1 [52] induced by the DTX [53].

Cumulative evidences support the perspective that oxidative and ER stress has a strong connection. Reactive oxygen species are produced as by-products during the protein folding process, impairing redox state, but also, impaired redox state leads to further ER stress since the protein folding process is dependent on redox homeostasis. Therefore, in order to assess if quenching of boosted ROS levels relief the observed ER stress, H9c2 cells were co-treated with N-Acetylcysteine, a ROS scavenger, previously reported as cardioprotective agent against anthracyclines induced toxicity [54]. Our data show that reduction of oxidative stress, associated with NAC pre-treatment, regulates changes in gene expression of CHOP and protein of ATF6 induced by DTX, but not in the other ERS proteins analysed. Our data indicate that the effects of omentin on ERS are not associated with the redox state [55].

**Fig 5:**
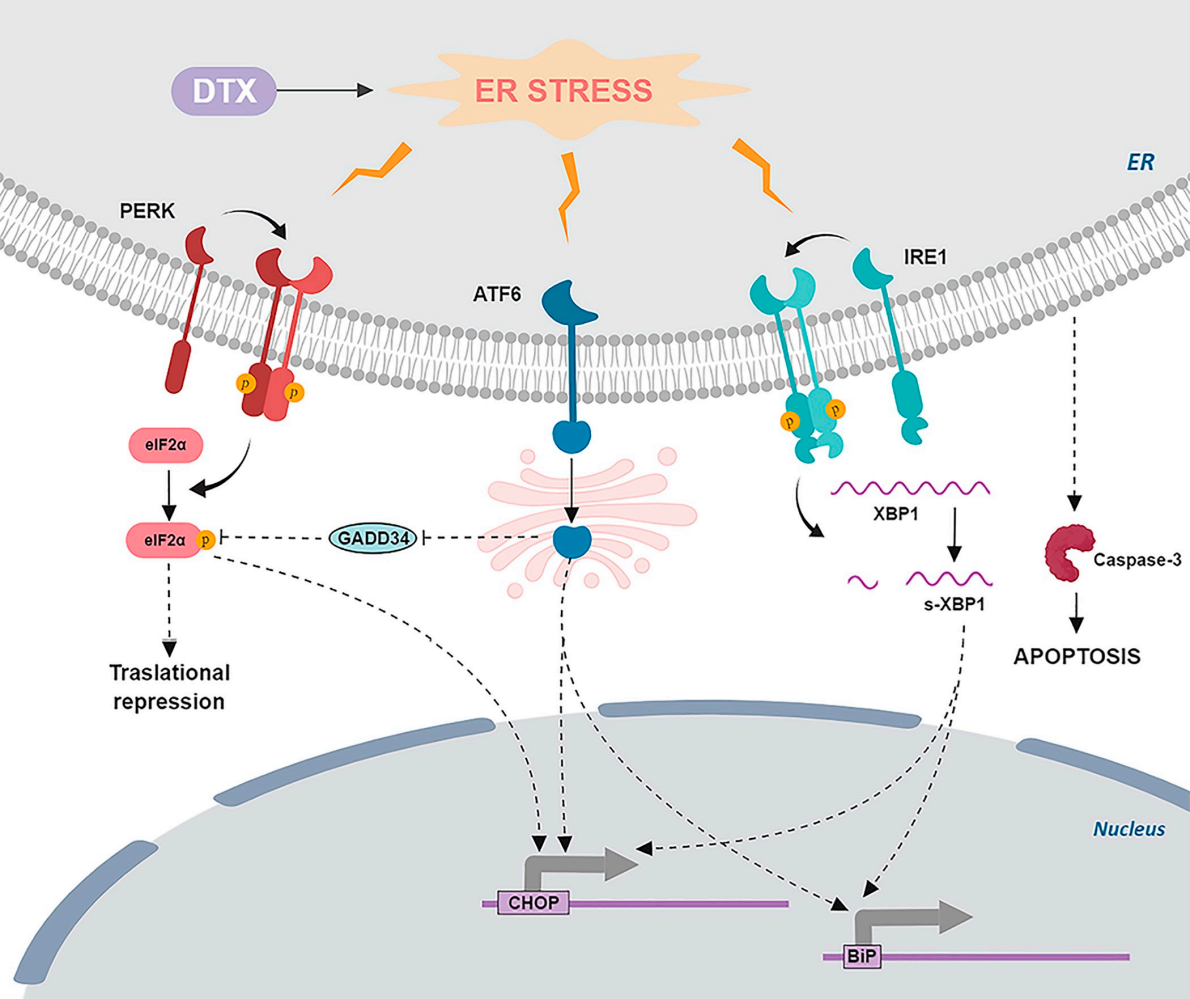
Proposed mechanism of DTX induced cardiotoxicity: Involvement of ER stress. DTX induces BIP, CHOP, ATF6 expression and reduces GADD34 expression increasing phosphorylated eIF2a levels. Unresolved ER stress will eventually lead to cell death (caspase-mediated apoptosis). Rounded ends indicate inhibitory pathways; arrows indicate stimulatory pathways.

In summary, our data showed that DTX has a direct effect on cardiomyoblasts, increasing oxidative and ERS and consequently cell death, suggesting that it could play a direct role in the development of myocardial toxicity. Our data does not clarify whether OMT counteracts or prevents the onset of ERS, but clearly demonstrated that OMT, despite not reducing DTX-induced oxidative stress, counteracts ERS and prevents apoptosis (Fig 5). These data suggest that omentin treatment may contribute to decrease susceptibility to DTX-induced cardiotoxicity. The results obtained in this study will be useful to carry out mechanistic *in vivo* model to explore the possibility of using omentin as a treatment against the cardiotoxicity induced by docetaxel.

## Supporting information

S1 Fig Effect of NAC in ERS gene and protein expression in H9c2 treated with DTX. mRNA levels of (A) BIP, (B) CHOP, (C) ATF6, (D) XBP1, (E) GADD34 and (F) MCPIP determined by real-time RT-PCR. Protein levels of (G) ATF6α, (H) peIF2α/eIF2α ratio and (I) BIP determined by western blot. All experiments were performed in H9c2 cells treated with DTX after 24 hours and/or pre treated with NAC for 1 hour. Data represent the means ± SEM from at least three independent experiments. Statistical significance *, ** and ***p < 0.05, 0.01, and 0.001 vs. control; #, ##, ### p< 0.05, 0.01 and 0.001 DTX-OMT vs. DTX.
(TIF)

S1 Table Primers for real time qPCR analysis. (DOCX)

S2 Table Antibodies used in western blot analysis. (DOCX)

## References

[1] Kovesdy CP, Furth SL, Zoccali C, World Kidney Day Steering C. Obesity and kidney disease: hidden consequences of the epidemic. J Nephrol. 2017;30(1):1–10. 10.1007/s40620-017-0377-y 28214961

[2] Mahmood SS, Levy D, Vasan RS, Wang TJ. The Framingham Heart Study and the epidemiology of cardiovascular disease: a historical perspective. Lancet. 2014;383(9921):999–1008. 10.1016/S0140-6736(13)61752-3 24084292 PMC4159698

[3] Calle EE, Rodriguez C, Walker-Thurmond K, Thun MJ. Overweight, obesity, and mortality from cancer in a prospectively studied cohort of U.S. adults. N Engl J Med. 2003;348(17):1625–38. 10.1056/NEJMoa021423 12711737

[4] Picon-Ruiz M, Morata-Tarifa C, Valle-Goffin JJ, Friedman ER, Slingerland JM. Obesity and adverse breast cancer risk and outcome: Mechanistic insights and strategies for intervention. CA Cancer J Clin. 2017;67(5):378–97. 10.3322/caac.21405 28763097 PMC5591063

[5] Johnson CB, Davis MK, Law A, Sulpher J. Shared Risk Factors for Cardiovascular Disease and Cancer: Implications for Preventive Health and Clinical Care in Oncology Patients. Can J Cardiol. 2016;32(7):900–7. 10.1016/j.cjca.2016.04.008 27343745

[6] Guenancia C, Lefebvre A, Cardinale D, Yu AF, Ladoire S, Ghiringhelli F, et al Obesity As a Risk Factor for Anthracyclines and Trastuzumab Cardiotoxicity in Breast Cancer: A Systematic Review and Meta-Analysis. J Clin Oncol. 2016;34(26):3157–65. 10.1200/JCO.2016.67.4846 27458291 PMC5569689

[7] Mitra MS, Donthamsetty S, White B, Mehendale HM. High fat diet-fed obese rats are highly sensitive to doxorubicin-induced cardiotoxicity. Toxicol Appl Pharmacol. 2008;231(3):413–22. 10.1016/j.taap.2008.05.006 18674790

[8] Guenancia C, Hachet O, Aboutabl M, Li N, Rigal E, Cottin Y, et al Overweight in mice, induced by perinatal programming, exacerbates doxorubicin and trastuzumab cardiotoxicity. Cancer Chemother Pharmacol. 2016;77(4):777–85. 10.1007/s00280-016-2995-9 26914236

[9] Maruyama S, Shibata R, Ohashi K, Ohashi T, Daida H, Walsh K, et al Adiponectin ameliorates doxorubicin-induced cardiotoxicity through Akt protein-dependent mechanism. J Biol Chem. 2011;286(37):32790–800. 10.1074/jbc.M111.245985 21784858 PMC3173230

[10] Kazama K, Okada M, Yamawaki H. Adipocytokine, omentin inhibits doxorubicin-induced H9c2 cardiomyoblasts apoptosis through the inhibition of mitochondrial reactive oxygen species. Biochem Biophys Res Commun. 2015;457(4):602–7. 10.1016/j.bbrc.2015.01.032 25600813

[11] Tan YL, Zheng XL, Tang CK. The protective functions of omentin in cardiovascular diseases. Clin Chim Acta. 2015;448:98–106. 10.1016/j.cca.2015.05.019 26079253

[12] Smekal A, Vaclavik J. Adipokines and cardiovascular disease: A comprehensive review. Biomed Pap Med Fac Univ Palacky Olomouc Czech Repub. 2017;161(1):31–40. 10.5507/bp.2017.002 28228651

[13] Kataoka Y, Shibata R, Ohashi K, Kambara T, Enomoto T, Uemura Y, et al Omentin prevents myocardial ischemic injury through AMP-activated protein kinase- and Akt-dependent mechanisms. J Am Coll Cardiol. 2014;63(24):2722–33. 10.1016/j.jacc.2014.03.032 24768874

[14] de Souza Batista CM, Yang RZ, Lee MJ, Glynn NM, Yu DZ, Pray J, et al Omentin plasma levels and gene expression are decreased in obesity. Diabetes. 2007;56(6):1655–61. 10.2337/db06-1506 17329619

[15] Maeda K, Saigo C, Kito Y, Sakuratani T, Yoshida K, Takeuchi T. Expression of TMEM207 in Colorectal Cancer: Relation between TMEM207 and Intelectin-1. J Cancer. 2016;7(2):207–13. 10.7150/jca.13732 26819645 PMC4716854

[16] Berardi R, Caramanti M, Savini A, Chiorrini S, Pierantoni C, Onofri A, et al State of the art for cardiotoxicity due to chemotherapy and to targeted therapies: a literature review. Crit Rev Oncol Hematol. 2013;88(1):75–86. 10.1016/j.critrevonc.2013.02.007 23522920

[17] Olsen SR. Taxanes and COX-2 inhibitors: from molecular pathways to clinical practice. Biomed Pharmacother. 2005;59 Suppl 2:S306–10. 16507399 10.1016/s0753-3322(05)80052-6

[18] Gundersen GG, Cook TA. Microtubules and signal transduction. Curr Opin Cell Biol. 1999;11(1):81–94. 10047525 10.1016/s0955-0674(99)80010-6

[19] Todaro MC, Oreto L, Qamar R, Paterick TE, Carerj S, Khandheria BK. Cardioncology: state of the heart. Int J Cardiol. 2013;168(2):680–7. 10.1016/j.ijcard.2013.03.133 23639459

[20] Rohena CC, Mooberry SL. Recent progress with microtubule stabilizers: new compounds, binding modes and cellular activities. Nat Prod Rep. 2014;31(3):335–55. 10.1039/c3np70092e 24481420 PMC4167679

[21] Hescheler J, Meyer R, Plant S, Krautwurst D, Rosenthal W, Schultz G. Morphological, biochemical, and electrophysiological characterization of a clonal cell (H9c2) line from rat heart. Circ Res. 1991;69(6):1476–86. 1683272 10.1161/01.res.69.6.1476

[22] Kuznetsov AV, Javadov S, Sickinger S, Frotschnig S, Grimm M. H9c2 and HL-1 cells demonstrate distinct features of energy metabolism, mitochondrial function and sensitivity to hypoxia-reoxygenation. Biochim Biophys Acta. 2015;1853(2):276–84. 10.1016/j.bbamcr.2014.11.015 25450968 PMC4388199

[23] Kazama K, Usui T, Okada M, Hara Y, Yamawaki H. Omentin plays an anti-inflammatory role through inhibition of TNF-alpha-induced superoxide production in vascular smooth muscle cells. Eur J Pharmacol. 2012;686(1–3):116–23. 10.1016/j.ejphar.2012.04.033 22554771

[24] Hung CH, Chan SH, Chu PM, Tsai KL. Docetaxel Facilitates Endothelial Dysfunction through Oxidative Stress via Modulation of Protein Kinase C Beta: The Protective Effects of Sotrastaurin. Toxicol Sci. 2015;145(1):59–67. 10.1093/toxsci/kfv017 25634538 PMC4833034

[25] Mhaidat NM, Thorne R, Zhang XD, Hersey P. Involvement of endoplasmic reticulum stress in Docetaxel-induced JNK-dependent apoptosis of human melanoma. Apoptosis. 2008;13(12):1505–12. 10.1007/s10495-008-0276-8 18989785

[26] Mathur A, Abd Elmageed ZY, Liu X, Kostochka ML, Zhang H, Abdel-Mageed AB, et al Subverting ER-stress towards apoptosis by nelfinavir and curcumin coexposure augments docetaxel efficacy in castration resistant prostate cancer cells. PLoS One. 2014;9(8):e103109 10.1371/journal.pone.0103109 25121735 PMC4133210

[27] Renehan AG, Tyson M, Egger M, Heller RF, Zwahlen M. Body-mass index and incidence of cancer: a systematic review and meta-analysis of prospective observational studies. Lancet. 2008;371(9612):569–78. 10.1016/S0140-6736(08)60269-X 18280327

[28] Curigliano G, Cardinale D, Dent S, Criscitiello C, Aseyev O, Lenihan D, et al Cardiotoxicity of anticancer treatments: Epidemiology, detection, and management. CA Cancer J Clin. 2016;66(4):309–25. 10.3322/caac.21341 26919165

[29] Spallarossa P, Maurea N, Cadeddu C, Madonna R, Mele D, Monte I, et al A recommended practical approach to the management of anthracycline-based chemotherapy cardiotoxicity: an opinion paper of the working group on drug cardiotoxicity and cardioprotection, Italian Society of Cardiology. J Cardiovasc Med (Hagerstown). 2016;17 Suppl 1 Special issue on Cardiotoxicity from Antiblastic Drugs and Cardioprotection:e84–e92. 27183529 10.2459/JCM.0000000000000381 PMC4927325

[30] Cueva JF, Antolin S, Calvo L, Fernandez I, Ramos M, de Paz L, et al Galician consensus on management of cardiotoxicity in breast cancer: risk factors, prevention, and early intervention. Clin Transl Oncol. 2017;19(9):1067–78. 10.1007/s12094-017-1648-8 28342058 PMC5547178

[31] Brown SA, Sandhu N, Herrmann J. Systems biology approaches to adverse drug effects: the example of cardio-oncology. Nat Rev Clin Oncol. 2015;12(12):718–31. 10.1038/nrclinonc.2015.168 26462128

[32] Duarte CW, Lindner V, Francis SA, Schoormans D. Visualization of Cancer and Cardiovascular Disease Co-Occurrence With Network Methods. JCO Clinical Cancer Informatics. 2017(1):1–12. 10.1200/CCI.16.00071 30657376

[33] Shibata R, Ohashi K, Murohara T, Ouchi N. The potential of adipokines as therapeutic agents for cardiovascular disease. Cytokine Growth Factor Rev. 2014;25(4):483–7. 10.1016/j.cytogfr.2014.07.005 25066649

[34] Gu N, Dong Y, Tian Y, Di Z, Liu Z, Chang M, et al Anti-apoptotic and angiogenic effects of intelectin-1 in rat cerebral ischemia. Brain Res Bull. 2017;130:27–35. 10.1016/j.brainresbull.2016.12.006 28017783

[35] Maruyama S, Shibata R, Kikuchi R, Izumiya Y, Rokutanda T, Araki S, et al Fat-derived factor omentin stimulates endothelial cell function and ischemia-induced revascularization via endothelial nitric oxide synthase-dependent mechanism. J Biol Chem. 2012;287(1):408–17. 10.1074/jbc.M111.261818 22081609 PMC3249092

[36] Ogura T, Tanaka Y, Tamaki H, Harada M. Docetaxel induces Bcl-2- and pro-apoptotic caspase-independent death of human prostate cancer DU145 cells. Int J Oncol. 2016;48(6):2330–8. 10.3892/ijo.2016.3482 27082738 PMC4864052

[37] Iida S, Shimada J, Sakagami H. Cytotoxicity induced by docetaxel in human oral squamous cell carcinoma cell lines. In Vivo. 2013;27(3):321–32. 23606687

[38] Hickson J, Ackler S, Klaubert D, Bouska J, Ellis P, Foster K, et al Noninvasive molecular imaging of apoptosis in vivo using a modified firefly luciferase substrate, Z-DEVD-aminoluciferin. Cell Death Differ. 2010;17(6):1003–10. 10.1038/cdd.2009.205 20057500

[39] Wu S, Li Q, Du M, Li SY, Ren J. Cardiac-specific overexpression of catalase prolongs lifespan and attenuates ageing-induced cardiomyocyte contractile dysfunction and protein damage. Clin Exp Pharmacol Physiol. 2007;34(1–2):81–7. 10.1111/j.1440-1681.2007.04540.x 17201740

[40] Theccanat T, Philip JL, Razzaque AM, Ludmer N, Li J, Xu X, et al Regulation of cellular oxidative stress and apoptosis by G protein-coupled receptor kinase-2; The role of NADPH oxidase 4. Cell Signal. 2016;28(3):190–203. 10.1016/j.cellsig.2015.11.013 26631573 PMC4837949

[41] Kazama K, Okada M, Yamawaki H. A novel adipocytokine, omentin, inhibits platelet-derived growth factor-BB-induced vascular smooth muscle cell migration through antioxidative mechanism. Am J Physiol Heart Circ Physiol. 2014;306(12):H1714–9. 10.1152/ajpheart.00048.2014 24727494

[42] Staren'kii VP, Vasil'ev L, Nikitchenko Iu V, Uzlenkova NE, Dziuba VN, Medvedeva EP, et al [Effect of subtherapeutic doses of docetaxel (taxotere) on the efficacy of radiotherapy and pro-oxidant-antioxidant balance in rats with Guerin's carcinoma]. Radiats Biol Radioecol. 2003;43(6):640–6. 14963930

[43] Tabaczar S, Pieniazek A, Czepas J, Piasecka-Zelga J, Gwozdzinski K, Koceva-Chyla A. Quercetin attenuates oxidative stress in the blood plasma of rats bearing DMBA-induced mammary cancer and treated with a combination of doxorubicin and docetaxel. Gen Physiol Biophys. 2013;32(4):535–43. 10.4149/gpb_2013048 24067283

[44] Yang Z, Fong DW, Yin L, Wong Y, Huang W. Liposomes modulate docetaxel-induced lipid oxidization and membrane damage in human hepatoma cells. J Liposome Res. 2009;19(2):122–30. 10.1080/08982100802632649 19235543

[45] Zhang Z, Zhao L, Zhou Y, Lu X, Wang Z, Wang J, et al Taurine ameliorated homocysteine-induced H9C2 cardiomyocyte apoptosis by modulating endoplasmic reticulum stress. Apoptosis. 2017;22(5):647–61. 10.1007/s10495-017-1351-9 28229251

[46] Wang M, Kaufman RJ. Protein misfolding in the endoplasmic reticulum as a conduit to human disease. Nature. 2016;529(7586):326–35. 10.1038/nature17041 26791723

[47] Nishitoh H. CHOP is a multifunctional transcription factor in the ER stress response. J Biochem. 2012;151(3):217–9. 10.1093/jb/mvr143 22210905

[48] Tsuru A, Imai Y, Saito M, Kohno K. Novel mechanism of enhancing IRE1alpha-XBP1 signalling via the PERK-ATF4 pathway. Sci Rep. 2016;6:24217 10.1038/srep24217 27052593 PMC4823713

[49] Azfer A, Niu J, Rogers LM, Adamski FM, Kolattukudy PE. Activation of endoplasmic reticulum stress response during the development of ischemic heart disease. Am J Physiol Heart Circ Physiol. 2006;291(3):H1411–20. 10.1152/ajpheart.01378.2005 16617122 PMC1575464

[50] Sahin F, Celik HA, Aydin HH, Oktem G, Omay SB, Saydam G. The interaction between taxoids and serine/threonine protein phosphatase activities during taxan-induced apoptosis of HL 60 leukemic cells. Hematology. 2008;13(4):215–23. 10.1179/102453308X315997 18796247

[51] Avril T, Vauleon E, Chevet E. Endoplasmic reticulum stress signaling and chemotherapy resistance in solid cancers. Oncogenesis. 2017;6(8):e373 10.1038/oncsis.2017.72 28846078 PMC5608920

[52] Younce CW, Kolattukudy PE. MCP-1 causes cardiomyoblast death via autophagy resulting from ER stress caused by oxidative stress generated by inducing a novel zinc-finger protein, MCPIP. Biochem J. 2010;426(1):43–53. 10.1042/BJ20090976 19925454

[53] Qian DZ, Rademacher BL, Pittsenbarger J, Huang CY, Myrthue A, Higano CS, et al CCL2 is induced by chemotherapy and protects prostate cancer cells from docetaxel-induced cytotoxicity. Prostate. 2010;70(4):433–42. 10.1002/pros.21077 19866475 PMC2931415

[54] Fulbright JM, Egas-Bejar DE, Huh WW, Chandra J. Analysis of redox and apoptotic effects of anthracyclines to delineate a cardioprotective strategy. Cancer Chemother Pharmacol. 2015;76(6):1297–307. 10.1007/s00280-015-2879-4 26515054 PMC4651800

[55] Chong WC, Shastri MD, Eri R. Endoplasmic Reticulum Stress and Oxidative Stress: A Vicious Nexus Implicated in Bowel Disease Pathophysiology. Int J Mol Sci. 2017;18(4). 10.3390/ijms18040771 PMC5412355 28379196

