# Supplementary figures and images for: Omentin protects H9c2 cells against docetaxel cardiotoxicity
Source: PLoS One. 2019 Feb 22;14(2):e0212782. doi: 10.1371/journal.pone.0212782 (PMC6386316; doi:10.1371/journal.pone.0212782)

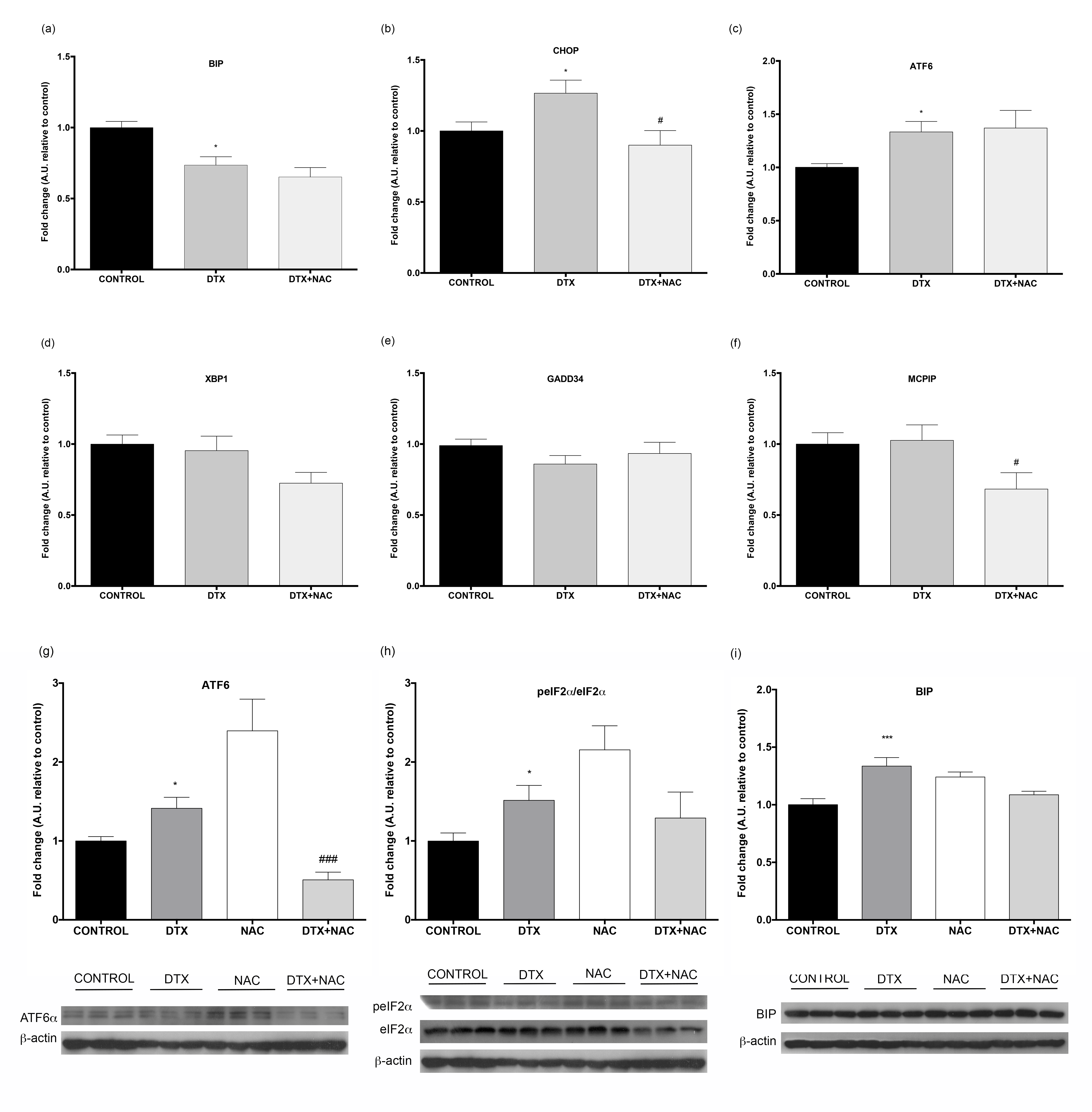

Supplement: S1 Fig — mRNA levels of (A) BIP, (B) CHOP, (C) ATF6, (D) XBP1, (E) GADD34 and (F) MCPIP determined by real-time RT-PCR. Protein levels of (G) ATF6α, (H) peIF2α/eIF2α ratio and (I) BIP determined by western blot. All experiments were performed in H9c2 cells treated with DTX after 24 hours and/or pre treated with NAC for 1 hour. Data represent the means ± SEM from at least three independent experiments. Statistical significance *, ** and ***p < 0.05, 0.01, and 0.001 vs. control; #, ##, ### p< 0.05, 0.01 and 0.001 DTX-OMT vs. DTX. (TIF) [file pone.0212782.s001.tif]
