# Supplementary material for: Omentin protects H9c2 cells against docetaxel cardiotoxicity
Source: PLoS One. 2019 Feb 22;14(2):e0212782. doi: 10.1371/journal.pone.0212782 (PMC6386316; doi:10.1371/journal.pone.0212782)
Supplement: S1 Table — (DOCX) [file pone.0212782.s002.docx]

**S1 Table.** Primers for real time qPCR analysis

| Gene | Primer-Forward | Primer-Reverse |
| --- | --- | --- |
| BiP | 5´-GAGGCGTATTTGGGAAAGAA-3´ | 5´-TCATGACATTCAGTCCAGCAA-3´ |
| CHOP | 5´-ACCACCACACCTGAAAGCA-3´ | 5´-AGCTGGACACTGTCTCAAAGG-3´ |
| ATF6 | 5´-GGACCAGGTGGTGTCAGAG-3´ | 5´-GACAGCTCTGCGCTTTGG-3´ |
| XBP1 | 5´-GCTGAGTCCGCAGCAGGT-3´ | 5´-ACAGGGTCCAACTTGTCCAG-3´ |
| GADD34 | 5´-GCTTTTGGCAAACCGAAC-3´ | 5´-TTCCAGTGCAGGACATGCT-3´ |
| MCPIP | 5´-TGGTCATCGACGGAAGCAAC-3´ | 5´-CCGCTCCAGAAACCAGTTCA-3´ |
| CAT | 5′-GCGAATGGAGAGGCAGTGTAC-3´ | 5′-GAGTGACGTTGTCTTCATTAGCACTG-3´ |
| GPx | 5′-CTCTCCGCGGTGGCACAGT-3´ | 5′-CCACCACCGGGTCGGACATAC-3 |
| SOD1 | 5′-GCAGAAGGCAAGCGGTGAAC-3´ | 5′-TAGCAGGACAGCAGATGAGT-3 |
| NOX1 | 5´-CTGACAAGTACTATTACACGAGAG-3´ | 5´-CATATATGCCACCAGCTTATGGAAG-3´ |
| NOX2 | 5´-CTTTAGCATCCATATCCGCATT-3´ | 5´-GACTGGTGGCATTGTCACAATA-3´ |
| NOX4 | 5´-TGTTGGGCCTAGGATTGTGT-3´ | 5´-GTGCGGCACATGGGTAAAAG-3´ |
| 36B4 | 5´-GGCGTCCTCATTAGAGTGACA-3´ | 5´- TAGTTGGACTTCCAGGTCGC-3´ |
