# Supplementary material for: Omentin protects H9c2 cells against docetaxel cardiotoxicity
Source: PLoS One. 2019 Feb 22;14(2):e0212782. doi: 10.1371/journal.pone.0212782 (PMC6386316; doi:10.1371/journal.pone.0212782)
Supplement: S2 Table — (DOCX) [file pone.0212782.s003.docx]

**S2 Table.** Antibodies used in western blot analysis

| **Antibody** | **Manufacturer** | **Cat. No** | **Clonality** | **Host** | **Dilution** |
| --- | --- | --- | --- | --- | --- |
| Anti-Caspase 3 | Cell Signalling | 9662 | Polyclonal | Rabbit | 1:1000 |
| Atf6α | Santa Cruz Biotech. | sc166659 | Monoclonal | Mouse | 1:1000 |
| BIP | Santa Cruz Biotech. | sc376768 | Monoclonal | Mouse | 1:500 |
| eIF2α | Santa Cruz Biotech. | sc11386 | Polyclonal | Rabbit | 1:1000 |
| p-eIF2α | Santa Cruz Biotech. | sc10670 | Polyclonal | Rabbit | 1:500 |
| CAT (H9) | Santa Cruz Biotech. | sc271803 | Monoclonal | Mouse | 1:1000 |
| GPx-1/2 (B-6) | Santa Cruz Biotech. | sc133160 | Monoclonal | Mouse | 1:100 |
| SOD-1 (B-1) | Santa Cruz Biotech. | sc271014 | Monoclonal | Mouse | 1:500 |
| Anti-β-actin | Sigma-Aldrich | A5316 | Monoclonal | Mouse | 1:1000 |
| Anti-Rabbit IgG | Cell Signalling | 7074 | Polyclonal | Goat | 1:3000 |
| Anti-Mouse Immunoglobulins | DAKO | P0447 | Polyclonal | Goat | 1:5000 |
